# Does digital literacy affect farmers’ adoption of agricultural social services? An empirical study based on China Land Economic Survey data

**DOI:** 10.1371/journal.pone.0320318

**Published:** 2025-04-23

**Authors:** Yanfei Wang, Guandong Xu, Jie Cao, Yanchi Chen, Jia Wu

**Affiliations:** 1 Institute of Food and Strategic Reserves, Nanjing University of Finance and Economics, Nanjing, Jiangsu, China; 2 Research Facility of Data Science and Artificial Intelligence, The Education University of Hong Kong, Hongkong, China; 3 Faculty of Science and Engineering, Macquarie University, Sydney, Australia; Manipal Academy of Higher Education, INDIA

## Abstract

Agricultural social services (ASS) are crucial in alleviating resource constraints and advancing agricultural modernization. Using the data derived from the China Land Economic Survey (CLES2022), this study empirically investigates how digital literacy influences farmers’ adoption of ASS, employing both Probit and Propensity score matching (PSM) models. Additionally, it explores the mediating roles of long-term production vision and part-time employment degrees in this relationship. The findings are: (1) digital literacy exhibited a statistically significant positive effect on farmers’ adoption of ASS at the 1% significant level. Moreover, this impact varied among participants in technology training, different education levels, and varying levels of risk preference; (2) long-term production vision and part-time employment degrees act as mediators, enhancing the positive impact of digital literacy on farmers’ adoption of ASS. Based on these findings, recommendations have been developed to improve farmers’ digital literacy, promote the adoption of ASS, and enhance farmers’ long-term production vision as well as degree of part-time.

## 1. Introduction

Agricultural social services (ASS) have been instrumental in reducing farmers’ resource constraints, boosting grain production, and increasing income. ASS is a key support in bridging the gap between small farmers and modern agriculture, thereby advancing agricultural modernization [1,2]. In China, the total revenue of ASS market has reached 23.9 billion dollars, the total number of various ASS organizations is 1.041 million, of which 89.39 million serve small farmers [3]. However, problems such as small-scale farmland and high land fragmentation have weakened farmers’ willingness to adopt ASS in the practice [4]. The study has pointed out that the acceptance degree of certain ASS by farmers is currently low [5], which, to some extent, restricts the sustainable development of ASS. How to encourage farmers to adopt ASS has become an urgent task to promote the healthy development of ASS [6]. The digital economy, driven by digital knowledge and information as key production factors, is advancing rapidly [7]. It promotes the integration of ASS with digital services while significantly improving the digital literacy of rural residents, providing new possibilities for farmers to adopt ASS [8]. Therefore, it is important to investigate the connection between digital literacy and farmers’ adoption of ASS to effectively increase the uptake of these services among all farmer groups and to broaden the application of behavioral theories in the context of the digital economy and ASS.

Can digital literacy effectively facilitate the real-time adoption of ASS by farmers? If so, what mechanisms underlie this effect? Additionally, how does the influence of digital literacy on farmers’ adoption of social services differ based on various individual characteristics? While previous studies provide useful insights, there is room for improvement in both research perspective and content. Most of the relevant literature explores the impact of digital literacy on farmers’ green production [9,10], income poverty reduction [11], employment quality [12], etc. Research on the impact of digital literacy on farmers’ adoption of ASS is currently limited. A detailed analysis of how digital literacy affects farmers’ adoption of ASS and the underlying mechanisms is essential for fully understanding the role of digital literacy in boosting the uptake of these services. This research holds significant practical importance in accelerating agricultural modernization. Thus, this study explores farmers’ adoption of ASS through the lens of digital literacy, utilizing survey data from the China Land Economic Survey (CLES2022). It aims to offer insights for promoting farmers’ adoption behavior of ASS and advancing agricultural modernization.

The rest of this paper is organized as follows: A literature review is provided in the second section. The research hypothesis is presented in the third section. The fourth section covers the research design, followed by an analysis of the empirical results in the fifth section, and further discussions are presented in the sixth section. The seventh section is policy recommendations.

## 2. Literature review

Research on how digital literacy affects farmers’ adoption of ASS is closely linked to the following research topics. Firstly, the results related to the measurement of farmers’ digital literacy and its spillover effects. Research on assessing farmers’ digital literacy remains somewhat limited. Scholars typically develop evaluation index systems that encompass a range of dimensions, such as information and data management, communication and collaboration proficiency, digital content creation, cybersecurity awareness, and problem-solving skills, among other factors [13,14]. For instance, Bai evaluated farmers’ digital literacy by analyzing their skills in computer usage, preferred payment methods, online communication expenses, online shopping habits, and online borrowing practices [15]. Some research indicates that digital literacy involves elements like digital ethics, participation, and critical abilities [16], further elaborating on its definition. Zhao et al. found that internet usage can help Chinese farmers reduce pesticide use [17]. Karakara and Daudu investigated the impact of digital literacy on the financial inclusion of farmers in Côte d’Ivoire and Nigeria [18]. Dzator et al. examined the mechanisms through which increased information and communication technology contributes to poverty reduction in 44 sub-Saharan African (SSA) countries [19]. Xie highlighted that the popularization of smartphones has a “two-sided” effect on the intensity of pesticide usage [20]. Overall, as global digital technology continues to advance, the concept of digital literacy has evolved from a superficial understanding to a deeper one.

Secondly, the research related to measurement of farmers’ adoption behavior of ASS and factors influencing it. There is a greater abundance of research results on evaluating the measurement of farm households’ adoption of ASS, and most of the literature on the measurement of this adoption behavior is attributed to the two choices of “whether or not to adopt” (dummy variables) [21,22], or characterized by the number of different segments of social services purchased by farmers [23]. Pagliacci et al. through a case study of northeastern Italy farmers, finds that farming, environmental, accessibility, policy factors all have impact on farmers adoption of climate-smart agricultural services [24]. Suvedi et al. found that training, membership in farmer groups, and off-farm employment are all associated with Nepalese farmers’ decisions to adopt agricultural services [25]. A dynamic context is provided for scholars’ future research on farmers’ adoption of ASS.

Thirdly, findings on the relationship between digital literacy and farmers’ adoption of ASS. Although digital literacy has gradually become a hot topic of scholars’ attention in recent years, there still few results on the correlation between digital literacy and farmers’ ASS adoption behavior [26,27]. Several studies have hinted at potential mechanism of how digital literacy influences farmers’ adoption of ASS when analyzing the impact of digital literacy on green production and food loss reduction behaviors of farmers [28,29]. Some scholars have argued that digital literacy can influence farmers’ adoption of green production technologies and their implementation of fertilizer and pesticide reduction. This influence occurs through various channels, such as promoting farmers’ access to and understanding of relevant information and knowledge, expanding access to credit, facilitating the implementation of long-term production visions, and increasing pressures for environmental protection [30,31]. These insights have provided scholars with directional guidance for further exploring the connection between digital literacy and farmers’ ASS adoption behavior.

## 3. Hypothesis

### 3.1. The direct impact of digital literacy on farmers’ adoption of agricultural social services

First, the “information effect” should be brought into play to enhance farmers’ knowledge of and willingness to adopt ASS. Information search theory shows that digital literacy can broaden the sources for farmers to obtain ASS and improve their access efficiency. Farmers with higher digital literacy can use agricultural service apps, mobile application platforms, Instagram, YouTube, Facebook, TikTok, and other internet tools to gain online skill training and interact with ASS providers. In addition, digital literacy enhances farmers’ social interaction, thereby reducing service uncertainty. They can also conveniently understand the actual effects and risks of adopting ASS through online social media, and communicate in real time with large households and capable growers, etc., which promotes knowledge sharing and technology complementation among farmers and enhances the precision of choosing these service providers [32–34]. The “information effect” fosters increasement of farmers’ cognitive level, pushes farmers to change the traditional smallholder production concept of self-farming, and enhances their willingness to adopt agricultural social services.

Secondly, the “transaction cost effect” is played to decrease the deviation between farmers’ willingness to adopt ASS and their adoption behavior. Although a considerable portion of farmers have the will to adopt agricultural social services, but not really implemented, which indicates that there is a certain level of contradiction between farmers’ intentions and actual behavior [35]. According to the theory of contract farming, farmers with high digital literacy can conveniently use the competitive bargaining power brought by the transparency of the digital service platform to compare the price and quality of ASS projects [36], effectively reducing the negotiation cost of farmers [37]. In addition, farmers that have high digital literacy are more inclined to utilize online platform to monitor the process of ASS online to ensure that the service provider uses the agreed-upon machinery or technology to carry out the services, which solves the problem of difficult to observe the quality of the services and the possible opportunistic behavior of the service provider [38], and effectively reduces the cost of monitoring the quality of the services. The reduction of transaction costs, such as negotiation costs and monitoring costs, will weaken the risk expectations of farmers in using ASS and promote the transition from adaptation willingness to actual behavior.

Thirdly, being digitally literate boosts property values and income while making credit access easier. This, in turn, enhances farmers’ ability to purchase ASS. Digital literacy can cultivate and strengthen farmers’ awareness of the market economy, expand the value transformation path of farmers’ existing assets, improve the efficiency of their asset allocation, and increase their property income [39]. Additionally, digital literacy can boost farmers’ non-farm income by supporting the diversification of their family livelihoods [40,41]. Finally, digital literacy significantly enhances farmers’ engagement in digital financial practices, farmers can conveniently use digital financial tools such as ants borrowing, WeBank, Trust etc., weakening the geographical limitations and collateral thresholds of financial credit, and effectively alleviating financial constraints [42,43].

Therefore, digital literacy can encourage farmers to adopt ASS by exerting the “information effect” to enhance farmers’ awareness and willingness to adopt, exerting the “transaction cost effect” to reduce the discrepancy between willingness and actual behavior, promoting property value-added and income increase to strengthen farmers’ purchasing power. Consequently, hypothesis 1 is proposed:

H1: Digital literacy positively influences farmers’ adoption of ASS.

### 3.2. The mediation effect of long-term production vision in digital literacy affecting farmers’ adoption of agricultural social services

From one perspective, digital literacy can facilitate the long-term production concept of farmers. Under the smallholder economic model, many farmers have a deep-rooted view of interest that “paying attention to immediate and ignoring long-term”, which leads them to place greater emphasis on the immediate cost-benefit of adopting ASS and may even refuse to adopt ASS due to short-term “uneconomic” expectations. Digital literacy enables farmers to access and interpret information about consumer preferences and premium details for high-quality agricultural products through WeChat official accounts, agricultural service apps, and other online platforms. This understanding supports them in making more informed long-term decisions [28]. From the other perspective, the broaden of long-term production vision can facilitate farmers’ adoption of ASS. Farmers with broad long-term production vision consider the cost-benefit of production activities in a more abundant time period and are able to fully verify the effects of adopting ASS on improving productivity, household income, saving labor time and reducing hard work degree, thus fully enjoying the improvement of subjective and objective welfare effects brought by the adoption of ASS. This positive incentive is conveyed to more farmers through the internet and neighborhood effects, thus enhancing farmers’ uptake of ASS. Accordingly, hypothesis 2 is formulated.

H2: Long-term production vision mediates the impact of digital literacy on farmers’ adoption of ASS.

### 3.3. The mediation effect of part-time employment degree in digital literacy affecting farmers’ adoption of agricultural social services

**Fig 1:**
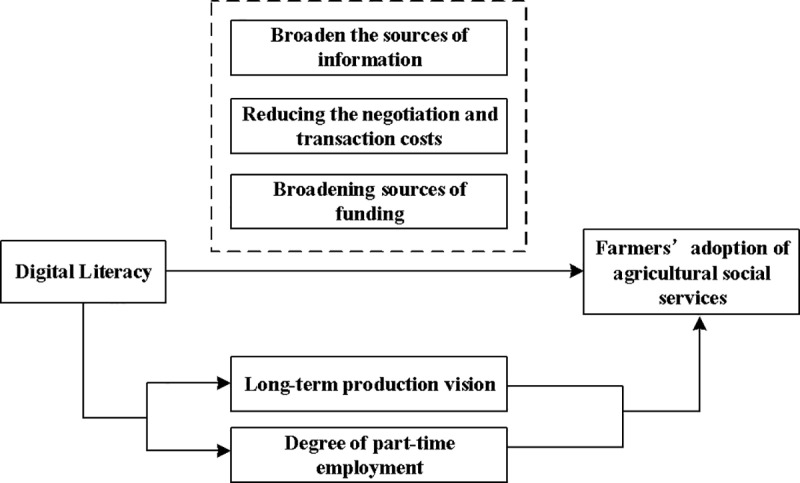
Intrinsic mechanism of digital literacy promotes farmers’ adoption of ASS.

On one hand, digital literacy can increase the extent of farmers’ involvement in part-time work. Higher digital literacy allows farmers to overcome spatial and temporal barriers to information, keeping them informed about domestic and international economic trends and industry developments. This reduces the shortage of entrepreneurial and employment resources [44,45], thus enhancing farmers’ extent of part-time employment. On the other hand, a higher level of part-time employment can encourage farmers to utilize ASS. Part-time farmers engaged in non-agricultural employment will lead to a lack of labor on family farms. Given the existing land transfer mechanisms in China, farmers face challenges in altering the structure of food cultivation in the short term. Purchasing ASS has become a realistic choice to solve the shortage of agricultural labor. In addition, non-farm entrepreneurship and employment helps to increase the total income of farmers and makes them more capable of purchasing agricultural social services. Therefore, hypothesis 3 is proposed. The main mechanism are shown in Fig 1.

H3: Part-time employment degree mediates the impact of digital literacy on farmers’ adoption of ASS.

## 4. Research design

### 4.1. Data source

Given the availability of data and the focus of the research, this paper adopts the 2022 China Land Economic Survey (CLES) data released by Nanjing Agricultural University. The CLES survey utilized the PPS sampling method and covered several prefecture-level cities in Jiangsu Province, which is broad and representative. A total of 1,203 questionnaires were collected from farmers in 24 administrative villages, 12 research districts and counties from six prefectural-level cities in Jiangsu Province. The CLES data covers information on the basic characteristics of farm households, production situation, rural infrastructure, etc., which is somewhat specialized and targeted. Due to the characteristics of the survey data, the CLES survey data may be subject to recall bias. When respondents answer questions related to past behaviors or events, inaccuracies or omissions in their memory may affect the truthfulness and accuracy of their responses. During the data processing, we first identified and selected relevant variables, including those reflecting digital literacy, farmers’ adoption of ASS, and control variables, based on the study objectives and the CLES questionnaire item settings. Subsequently, samples with missing values and outliers in the relevant variables were removed. After these steps, a total of 1,050 valid samples were obtained for this paper.

### 4.2. Selection of variable

#### 4.2.1. Explained variable.

Farmers’ adoption behavior of ASS. The CLES data have examined in detail of the pre-production services (seed supply, soil testing and fertilization, crop cultivation and management), mid-production services (pest control technology, mechanized production technology, energy-saving and efficient facility-based agricultural technology, water-saving irrigation technology, disaster prevention and mitigation technology), and post-production services (agricultural product processing, packaging, and preservation technology, comprehensive utilization of crop residue technology, clean renewable energy technology, agricultural policy information service, agricultural product market information service, and agricultural credit and financial services) involved in the agricultural production activities of farmers. Given the complexity of agricultural production and the need for accurate data, this paper uses the ratio of adoption for the aforementioned 16 types of ASS to analyze and quantify farmers’ adoption levels of these services, which is specifically expressed as follows:


$$ASSi=\frac{Sij}{16}$$ (1)

Where ASSi is the extent to which each farmer adopts ASS, and Sij is the number of categories of the 16 ASS adopted by *i* farmer. Given that each category of agricultural social services is related to agricultural production and marketing, the average weight is calculated as $\frac{1}{16}$.

#### 4.2.2. Core explanatory variable.

Digital literacy (DL). Reddy et al. [46], and Gong et al. [47] defined digital literacy of farmers as reflecting the ability of individual farmers to securely and effectively acquire, use, communicate, manage, evaluate, and create information or data by adopting digital technologies or through digital devices. In this paper, digital literacy level of farmers is measured by indicators such as accessibility of digital technology application (whether farmers utilize devices such as cell phones of computers for internet access), depth of digital technology usage (farmers’ capacity to leverage digital technology for accessing information and financial services, etc.), etc., and digital literacy indexes are synthesized by entropy value method. The specific indicator settings are shown in Table 1.

**Table 1:** Digital literacy assessment questions.

Typology	Index	Specific Assessment Questions	Weight
Digital technology accessibility	Number of cell phones	Number of cell phones you have	0.0504
	Number of computers	Number of computers you have	0.3115
Depth of digital technology usage	Digital information literacy	Information access, 1 = Basically access information through non-web channels, 2 = Mainly access information through non-web channels and less through web channels, 3 = There is not much difference in the proportion of information accessed through web channels and non-web channels, 4 = Mainly access information through web channels and less through non-web channels, 5 = Basically access information through web channels	0.2891
	Digital financial literacy	Daily payment transaction tools, 1 = Basically make payment through cash, 2 = Mainly make payment through cash and less through internet, 3 = The proportion of cash payment and internet payment is not less difference, 4 = Mainly make payment through the internet and less through cash, 5 = Basically make payment through the internet	0.3490

#### 4.2.3. Control variables.

Drawing on existing research, this paper incorporates control variables related to individual, household, farmland operation, and village location characteristics to reduce the impact of omitted variables on the estimation results [48]. The factor of individual dimension selects gender, age, education level, and health status. The factors for the household dimension select the total number of presence of cadres in the household. The control variables at the farmland management characteristics level include operation scale and degree of land fragmentation. Topographic features and transportation features choose to represent village-level characteristics. Although we attempted to capture the factors that might influence the effects of digital literacy on farmers’ adoption of ASS, there could still be unobserved factors, such as household social networks and individual psychology, that may introduce bias into the results. To address this issue, we conducted an endogeneity test. The specific meanings and assignments of control variables are shown in Table 2.

**Table 2:** Descriptive statistics.

Variable	Symbols	Specific Assessment Questions	Mean Value	Standard Deviation
Farmers’ adoption behavior of ASS	*ASS*	Extent of farmers’ adoption of ASS	0.0307	0.0765
Digital literacy	*DL*	Entropy method weighted average synthetic composite value	0.2807	0.2596
Gender	*Gender*	Male = 1; Female = 0	0.9057	0.2924
Age	*Age*	Farmer’s age	63.1438	10.0246
Education level	*Edu*	Length of schooling	7.4552	3.7577
Health status	*Health*	1 = Incapacitated; 2 = Poor 3 = Medium; 4 = Good; 5 = Excellent	3.8533	1.1624
Number of family members	*Rsi*	Actual number of family members	3.0752	1.5643
Identity in village	*Cadre*	Whether there are cadres in the family. 1 = Yes; 0 = No	0.1800	0.3844
Farmland scale	*Caa*	Contracted land area/ Mu	8.3369	19.5341
Degree of land fragmentation	*Ncp*	Number of plots of contracted land	3.6652	2.9231
Topographic characteristics	*Tf*	Topographic features of the village: 1 = Plains; 2 = Hills; 3 = Mountains	1.2486	0.4324
Transportation characteristics	*Vnhe*	Distance from the village committee to the nearest highway entrance	12.7982	11.1296
Long-term production vision	*Pd*	1 = Focusing only on current returns; 2 = Focusing on both current and future returns; 3 = Focusing only on future returns	1.7039	0.6924
Part-time employment degree	*Dpte*	Number of days of non-farm work performed by farmers in the last year	74.7767	118.8717

Table 2 shows the distribution of control variables, emphasizing individual characteristics among farmers. Male farmers account for 90.57% and female farmers account for 9.43%, which shows that men are still the main labor force and economic decision makers of the family in current agricultural production; young and middle-aged farmers account for 5.05%, middle-aged farmers account for 29.52%, and elderly farmers account for 65.43%, which shows that middle-aged and older individuals are the main labor force in agricultural production; 37.81% of farmers have an education level of junior school or lower, 41.71% have completed junior high school, and 20.48% have received senior high school education or higher, which shows that the interviewed farmers generally have a low level of education; and 84.67% of farmers reported medium to good physical health. At the level of family characteristics, the interviewed farmers’ family are mainly composed of more than three persons; only 18% of them have village cadres in their family. At the level of farmland operation characteristics, the typical cultivated area for the surveyed farmers is 8.34 mu, and 81.05% of them have a planting area smaller than the average, with an average of 3.67 plots, which shows that China is still facing the problem of “big country, small farmers”. In terms of village characteristics, 75.14% of the villages have plain topography, and most of the villages are close to highways, making transportation more convenient.

#### 4.2.4. Mediating variables.

This paper examines how digital literacy influences farmers’ adoption behavior of ASS, focusing on the implementation of long-term production vision (Pd) and the part-time employment degree (Dpte). Long-term production vision is measured by whether farmers focus on current or future return; and part-time employment degree is measured by the number of days farmers engaged in non-farm work (see Table 2).

### 4.3. Model construction

#### 4.3.1. Regression model.

To evaluate how digital literacy affects farmers’ adoption of ASS, the paper develops the following model:


ASSi=α0+α1DLi+α2Xi+εi (2)

In Equation (2), ASSi represents the extent to which individual farmers adopt ASS; DLi indicates the digital literacy of the *i* farmer; Xi includes the relevant control variables related to the farmers’ individual attributes, household details, farmland management and village characteristics; α0 is constant; α1 and α2 is the parameter to be estimated; εi is a random error term.

Second, the ordinary least squares regression may have estimation bias due to the possible endogeneity problem. To enhance the reliability of the research findings, this paper develops the following ordered probit model, building upon previous studies [49,50].


ASSi=α0+α1DLi+α2Xi+εi (3)

Assuming a μ∼N(0,1) distribution, the ordered probit model can be expressed as:


$$P(ASS=0 |x)=P(ASS^{*}\leq r0 |x)=\varphi (r0-\alpha 1DLi-\alpha 2Xi)$$

$$P(ASS=1 |x)=P(r0<ASS^{*}\leq r1 |x)=\varphi (r1-\alpha 1DLi-\alpha 2Xi)-\varphi (r0-\alpha 1DLi-\alpha 2Xi)$$

$$P(ASS=16 |x)=P(r15\leq ASS^{*} |x)=1-\varphi (r15-\alpha 1DLi-\alpha 2Xi)$$ (4)

In Equation (4), r0, r1 … r15 are the parameters to be estimated; the values of ASS are 0,1,2,…16, respectively indicating that farmers “did not adopt ASS” to “adopt 16 ASS”; *φ* is the cumulative density function of standard normal distribution. By constructing the likelihood function of each farmer’s adoption of ASS, the model parameters were estimated by the great likelihood method. The ordered probit model is specifically designed for analyzing ordinal categorical explained variables that are ordered but not equidistant. It’s worth noting that the ordered probit model may encounter issues related to omitted variables and endogeneity. To address these concerns, we consider using the propensity score matching model in conjunction with robustness tests.

Finally, in reference to Rosenbaum [51], propensity score matching was employed to address the issues related to self-selection. The propensity score matching method uses “counterfactual” inference to compare with the observed group of digitally literate farmers to achieve a reliable estimate of the average treatment effect (ATT), which is calculated as follows:


$$ATT=E(Y1i |Ii=1)-E(Y0i |Ii=1)$$ (5)

Where Y1i represents the adoption of ASS by digitally literate farmers, and Y0i represents the adoption of ASS when farmers who are not digitally literate are assumed to be digitally literate. It’s worth noting that the propensity score matching model may face issues related to matching quality. To mitigate this concern, we applied robustness tests to enhance the reliability of the results.

#### 4.3.2. Mediating effect model.

Since it is difficult to ensure the exogeneity of the mediating variables in the traditional mediation effect model, there are problems such as endogeneity bias and low efficacy of statistical tests, with reference to the mediation effect test methods of Jiang [52] and Yang et al. [53], this paper constructs the linear regression equations for the explanatory variable of digital literacy (DL) on the mechanism variables of long-term production vision (Pd) and part-time employment degree (Dpte) respectively. β0 and λ0 are constant terms; β1, β2, λ1, λ2 are parameters to be estimated; εi is the random error term.


Pdi=β0+β1DLi+β2Xi+εi (6)

Dptei=λ0+λ1DLi+λ2Xi+εi (7)

## 5. Analysis of the empirical findings

### 5.1. Benchmark regression results

Table 3 presents the regression results examining the impact of digital literacy on farmers’ adoption of ASS. The core explanatory variable consistently shows positive and significant regression coefficients, regardless of whether control variables are included, indicating that digital literacy promotes the adoption of these services. Regression (1) in Table 3 reveals that, after accounting for regional factors, the estimated coefficient for digital literacy’s effect on service adoption is 0.0726, significant at the 1% level, which means that for every one-unit increase in digital literacy, farmers’ adoption of ASS increases by 0.0726 unit. Regression (2) further adjusts for individual characteristics, family traits, farmland operations, and village attributes, with the coefficient for digital literacy’s impact on adoption estimated at 0.0562, also significant at the 1% level, which means that for every one-unit increase in digital literacy, farmers’ adoption of ASS increases by 0.0562 unit. This suggests that digital literacy can mitigate market information asymmetry and transaction costs, expand information and employment opportunities for farmers, and ultimately enhance their income and purchasing power, thereby facilitating their adoption of ASS.

**Table 3:** Regression results.

Variable	Benchmark Regression Model		Oprobit	
	(1)	(2)	(3)	(4)
	ASS	ASS	ASS	ASS
*DL*	0.0726***	0.0562***	1.2592***	1.0909***
	(0.0109)	(0.0125)	(0.1540)	(0.1978)
Control variables	No	Yes	No	Yes
Control region	Yes	Yes	Yes	Yes
_Cons	0.0020***	-0.0015		
	(0.0060)	(0.0369)		
N	1050	1050	1050	1050
R^2^	0.0652	0.0935		
Pseudo R^2^			0.0373	0.0600
Wald chi-squared statistic			66.85***	107.52***
Log pseudolikelihood			-810.9486	-791.8384

Standard errors are in brackets, * p <  0.1; **p <  0.05; ***p <  0.01.

### 5.2. Robustness test and endogenous treatment

To ensure the robustness of the estimation results, this section employs three methods for robustness testing and endogeneity analysis. The first is to utilize the ordered probit model. To apply the ordered probit model, we replace the explained variable with ordinal variables ranging from 0 to 16, where 0 indicates that farmers “did not adopt ASS” and 16 represents the adoption of all 16 ASS. The test results of (3) and (4) in Table 3 show that the estimated coefficients of the impact of digital literacy on the adoption of ASS by farmers are positive and significant regardless of the inclusion of control variables, which reconfirms that the improvement of digital literacy helps to promote the adoption of ASS by farmers.

Second, the propensity score matching method (PSM) is employed to address sample selection bias and establish the causal relationship between digital literacy and farmers’ adoption of ASS. The specific estimation results are shown in Table 4. From the estimation results, it can be found that digital literacy significantly promotes farmers’ adoption of ASS, and the estimation results of different matching methods have high consistency, which suggesting that the estimation results of this paper are robust.

**Table 4:** Propensity sore matching method results.

Matching Method	Experimental Group	Comparison Group	ATT	Standard Deviation	t-value
Radius matching	0.0398	0.0177	0.0221	0.0066	3.35
Nearest neighbor matching	0.0398	0.0177	0.0221	0.0066	3.35
Local linear regression matching	0.0398	0.0217	0.0181	0.0066	2.74
One-to-one matching	0.0399	0.0176	0.0223	0.0064	3.47

Third, the sample robustness test. Compared to younger people, older farmers are less capable of using digital devices and embracing digital technologies, and lack the initiative to subjectively accept digital information, so the correlation between their ASS adoption behavior and digital literacy may be weaker. Given that the World Health Organization defines people aged 60 and above as the elderly, of which 60-74 years old is the young elderly, and 75 years old and above is the general elderly, and the young elderly still have certain learning and acceptance ability, this paper excludes the sample of farmers aged 75 and above to re-run the regression, and the detailed estimation results are presented in Table 5. The estimation results of Table 5 (2) show that digital literacy has a significant positive impact on the adoption behavior of ASS of farmers. The conclusion that digital literacy positively influences farmers’ adoption of ASS is consistent with the findings of Zheng, et al. [54].

**Table 5:** Robustness test results.

Variable	(1)	(2)
	ASS	ASS
*DL*	0.0725***	0.0568***
	(0.0115)	(0.0131)
Control variables	No	Yes
Control region	Yes	Yes
_Cons	0.0028	-0.0096
	(0.0069)	(0.0426)
N	921	921
R^2^	0.0609	0.0910

Standard errors are in brackets, * p <  0.1; **p <  0.05; ***p <  0.01.

At this point, this paper concludes that the hypothesis 1 is valid.

### 5.3. Mediation effect test

The results confirm that digital literacy plays a significant role in increasing farmers’ adoption of ASS. This section further tested whether long-term production vision and the level of part-time employment play a mediating role in digital literacy in promoting farmers’ utilization of ASS. The estimation results of (1) and (2) in Table 6 show that digital literacy has a significant positive effect on farmers’ implementation of long-term production vision, regardless of whether control variables are included or not. After accounting for regional differences, the results from regression (1) in Table 6 indicate that the estimated coefficient for the impact of digital literacy on farmers’ long-term production vision is 0.4343, significant at the 1% level. Conversely, regression (2) in Table 6, which includes additional control variables, shows an estimated coefficient of 0.2405, significant at the 5% level. The reason why digital literacy can promote the implementation of long-term production vision of farmers may be that the production decisions of farmers are not entirely based on rationality, and their decisions may be affected by several factors, such as risk perception, perception of welfare effects, cognitive preferences, social influence, etc. Improving digital literacy facilitates farmers’ ability to gather and use information, encouraging them to make more informed and rational long-term decisions. The results from regressions (3) and (4) in Table 6 demonstrate that digital literacy positively and significantly impacts the level of part-time employment among farmers, with significance at the 1% level, regardless of the inclusion of control variables. The reason why digital literacy can enhance the level of part-time employment of farmers may be that the enhancement of digital literacy expands the information channels and social resources of farmers, provides farmers with more opportunities for self-employment and working outside, lowers the threshold of part-time employment of farmers. At this point, the two mediation mechanisms proposed in this paper have been preliminarily confirmed.

**Table 6:** Estimation results of mediation effects.

Variable	(1)	(2)	(3)	(4)
	Pd	Pd	Dpte	Dpte
*DL*	0.4343***	0.2405**	0.4681***	0.2448***
	(0.0810)	(0.1050)	(0.0397)	(0.0452)
Control variables	No	Yes	No	Yes
Control region	Yes	Yes	Yes	Yes
_Cons	1.4419***	1.6060***	0.1116***	0.2628 *
	(0.0570)	(0.3102)	(0.0309)	(0.1365)
N	1050	1050	1050	1050
R^2^	0.0331	0.0436	0.1663	0.2407

Standard errors are in brackets, * p <  0.1; **p <  0.05; ***p <  0.01.

The above mechanism is established on the premise that long-term production vision and part-time employment degree have impact on ASS adoption. To ensure robustness, this paper follows the approach of Yang et al. [53], by treating ASS adoption as the dependent variable and the mediation variable as the independent variable in the regression analysis. The results in Table 7 (1) indicate that implementing a long-term production vision has a positive and statistically significant effect on farmers’ adoption of ASS, with significance at the 5% level. The possible explanation is that the implementation of long-term production vision can enable farmers to measure and compare the cost-benefit of their production activities in a more abundant time period, and fully experience the improvement of subjective and objective welfare effects resulting from the adoption of ASS, thereby increasing the extent of their adoption of ASS. The estimation results in Table 7 (2) show that the positive effect of increasing part-time employment on farmers’ adoption behavior of ASS is statistically significant at the 5% level. Possible explanations are that farmers with a high degree of part-time employment have a stronger willingness to adopt ASS because of the opportunity cost of commuting during busy times; at the same time, farmers with a high degree of part-time employment have higher off-farm incomes, higher risk-taking ability, and the experience of working outside of the farm may improve their cognitive ability, which may increase the degree of their adoption of ASS. These results further strengthen the reliability of the mediation mechanism proposed in this paper.

**Table 7:** Regression results of mediation variables’ influence on adoption of ASS.

Variable	(1)	(2)
	ASS	ASS
*Pd*	0.0068**	
	(0.0034)	
*Dpte*		0.0188**
		(0.0084)
Control variables	Yes	Yes
Control region	Yes	Yes
_Cons	0.0018	0.0051
	(0.0090)	(0.0256)
N	1,050	1,050
R^2^	0.0445	0.0681

Standard errors are in brackets, * p <  0.1; **p <  0.05; ***p <  0.01.

At this point, this paper concludes that the hypothesis 2 and 3 are valid.

## 6. Further discussions

### 6.1. Heterogeneity analysis

This section analyzes heterogeneity in terms of whether farmers have received agricultural technology training, education level of farmers, and risk preferences of farmers. The first heterogeneity analysis is based on whether farmers have received training in agricultural technology. The presence or absence of training may affect farmers’ awareness and ability to embrace new technologies, which in turn leads to some variation in the impact of digital literacy on farmers’ adoption of ASS. The estimation results in Table 8 (A) show that regardless of whether or not farmers have received agricultural technology training, digital literacy has a positive and significant effect on the adoption of ASS in both groups. The possible reason why digital literacy has a more significant effect on the adoption behavior of farmers trained in agricultural technology may be that those who have received training have relatively broader vision and stronger understanding ability.

**Table 8:** Estimation results of heterogeneity test.

A: Whether farmers have received training in agricultural technology			
Variable	Have been trained	Have not been trained	
*DL*	0.0717***	0.0235**	
	(0.0231)	(0.0092)	
Control variables	Yes	Yes	
Control region	Yes	Yes	
_Cons	-0.0388	-0.0056	
	(0.0780)	(0.0272)	
N	455	595	
R^2^	0.1074	0.0474	
B: Education level			
Variable	Lower education level	Middle education level	Higher education level
*DL*	0.0555**	0.0612***	0.0365
	(0.0256)	(0.0181)	(0.0233)
Control variables	Yes	Yes	Yes
Control region	Yes	Yes	Yes
_Cons	-0.0185	-0.0647	0.0687
	(0.0411)	(0.0546)	(0.0918)
N	397	438	215
R^2^	0.0853	0.0634	0.2060
C: Risk preference level			
Variable	High-risk preference	Middle-risk preference	Low-risk preference
*DL*	0.0475	0.0505	0.0550***
	(0.0755)	(0.0392)	(0.0118)
Control variables	Yes	Yes	Yes
Control region	Yes	Yes	Yes
_Cons	0.1900	0.1633	-0.0586 *
	(0.2564)	(0.1009)	(0.0333)
N	60	180	810
R^2^	0.2007	0.2784	0.0820

Standard errors are in brackets, * p <  0.1; **p <  0.05; ***p <  0.01.

The second heterogeneity analysis is based on farmers’ education level. Education level influences farmers’ information sources and their ability to apply digital technology, thereby affecting how digital literacy impacts their adoption of ASS. This paper classifies farmers into three education categories: junior school and below (lower education level), junior high school (middle education level), and senior high school and above (higher education level) and excludes education level as a control variable in the regression analysis. The estimation results in Table 8 (B) reveal that digital literacy positively and significantly influences ASS adoption among farmers with low and medium education levels, but this effect is not significant for those with high education levels. The possible explanation is that farmers with low and medium education levels have fewer information channels and weaker digital technology application capabilities. As digital literacy improves, it can broaden their information access channels and enhance their digital technology application capabilities to a greater extent, thus significantly promoting social services adoption. On the contrary, highly educated farmers have wider access to information and better ability to apply digital technology, so the marginal effect of digital literacy diminishes.

The third heterogeneity analysis is based on farmers’ risk preferences. Farmers with different risk preferences may differ in acceptance of new things, which leads to disparity in the impact of digital literacy on farmers’ adoption of ASS. The paper classifies farmers’ risk preferences into three categories: high, medium, and low risk preference. The estimation results in Table 8 (C) indicate that digital literacy significantly positively affects ASS adoption among low-risk preference farmers but does not have a notable impact on those with high or medium-risk preferences. The possible reason is that low-risk preference farmers prefer to take agricultural production activities into their own hands and not trust other members for risk avoidance. Enhancing farmers’ digital literacy expands their knowledge of ASS and alleviates their concerns about transaction costs, which significantly encourages the adoption of ASS among low-risk farmers. In contrast, high- and medium-risk farmers are more inclined to experiment with new agricultural practices, so the additional benefit of increased digital literacy on their further adoption of ASS is relatively smaller.

## 7. Conclusions and policy recommendations

### 7.1. Conclusions

In the context of implementing the rural revitalization strategy, agricultural social services can help free farmers from manual labor and contribute to increasing their income. Therefore, it is crucial to emphasize the importance of and encourage the adoption of agricultural social services. This paper investigates how digital literacy affects farmers’ adoption of ASS and the mechanisms behind this influence. The theoretical analysis indicates that digital literacy not only plays a direct role in motivating farmer adoption of ASS, but also indirectly promotes farmers’ choice of these services by influencing their long-term production vision and part-time employment degree. This paper not only enriches the theoretical framework by examining the relationship between digital literacy and farmers’ adoption of ASS, but also offers valuable theoretical insights for the formulation of policies aimed at improving the promotion of agricultural social services and increasing farmers’ adoption of ASS.

The conclusions present as following: First, digital literacy facilitates farmers’ adoption of ASS. Secondly, the impact of digital literacy on farmers’ adoption of ASS is more pronounced across three types of farmer samples: those who have received agricultural technology training, those with medium or low educational levels, and those with low-risk preference. Third, long-term production visions and part-time employment degree have a mediating effect in promoting farmers’ adoption of agricultural socialization services.

The research in this manuscript has certain limitations that warrant further exploration. On one hand, due to data constraints, while efforts were made to capture a comprehensive measure of digital literacy, it may still fall short of fully representing its scope. Future research could refine the measurement system of digital literacy to better capture the differences across various demographic groups. On the other hand, the impact of digital literacy on farmers’ adoption of ASS may be influenced by several factors beyond the scope of this study, such as policy orientation, technological advancements, and other external variables. These factors may have affected the results but were not addressed in this study’s design. Future research could incorporate a broader range of external factors to more comprehensively investigate the relationship between farmers’ digital literacy and their adoption of ASS.

### 7.2. Policy recommendations

Based on these findings, the following policy recommendations are suggested. Firstly, various ways could be adopted to enhance farmers’ digital literacy, which will help to fully stimulate the role of digital literacy in promoting the adoption of ASS. The government should continuously increase the capital investment on rural digital infrastructure construction, and should actively establish and improve the rural digital literacy training system; encourage scientific research institutes, village committees, agricultural enterprises, large scale farmers and other subjects to focus on digital life, production, learning, innovation and other needs; make full use of media carriers to carry out typical demonstrations and perceptual guidance, and reasonably guide farmers to learn and improve their own digital literacy. Based on this, a fast and efficient mechanism for promoting and disseminating agricultural social services should be established. This can be achieved by leveraging internet tools such as WeChat official accounts, video accounts, and WeChat groups to help farmers access relevant information about the quality, price, and other aspects of agricultural social services more quickly and effectively. This approach can reduce farmers’ perception of uncertainty regarding agricultural social services, as well as lower transaction costs such as negotiation and supervision costs, thereby guiding and motivating farmers to actively adopt agricultural social services.

Secondly, it is important to focus on the mediating roles of long-term production vision and part-time employment, and fully leverage the potential of digital literacy to boost farmers’ adoption of ASS. On one hand, enhancing farmers’ digital literacy should help them evaluate the long-term cost-benefit of their production and operational activities, allowing them to fully appreciate the positive incentives for adopting ASS. On the other hand, it guides farmers to rely on digital literacy to improve their information acquisition ability, cognitive ability and professional skills, expand their entrepreneurial and employment resources, in order to enhance their degree of part-time employment and promote the active adoption of ASS.

Thirdly, the effect of digital literacy on ASS adoption could be enhanced based on analyzation of the heterogeneity characteristics. Based on the results of the heterogeneity test, the government should tailor policies according to the individual. Specifically, the government should build and improve the rural education system, focusing on strengthening agricultural technology training for small farmers, helping them improve their understanding of and adaptation to new technologies, as well as setting up demonstration villages and households for ASS to give full play to the “peer effect”.

Dataset: Nanjing Agricultural University, China Land Economic Survey, (2022).
